# Laboratory Information Management Systems- a survey

**DOI:** 10.1155/S1463924685000281

**Published:** 1985

**Authors:** K. Jones

**Affiliations:** Phase Separations Ltd Deeside Industrial Estate Clwyd Queensferry UK


Journal of Automatic Chemistry/*Journal of Clinical Laboratory, Automation*, Vol. 7, No. 3 (Jul–Sep 1985), pp. 136–140

### Laboratory Information Management Systems – a survey

**K. Jones**
*Phase Separations Ltd, Deeside Industrial Estate, Queensferry, Clwyd, UK*

The management of instruments, especially if centralized into one analytical environment, can demand commercial expertise as well as scientific knowledge. Considerable quantities of analytical data have to be acquired, manipulated, compiled and reported, in addition to such separate administrative functions as operational budgets, cost control, staff functions, external communications, materials stores and inventory, literature updating, and so on. This leads to an obvious parallel with more commercially related environments, such as stockbrokers, solicitor's offices and banks.

Commercial environments have adopted data transfer and computerized records as a matter of course. As pressures increase to minimize paperwork, and maximize efficiency, elimination of paper records is becoming a factor of survival in a highly competitive world. Analytical laboratories can be regarded as very little different from a purely commercial operation and yet only very recently have Laboratory Information Management Systems (LIMS) been offered to the scientific world. However, it appears that the systems currently on offer are not necessarily the optimum solution for many laboratories.

### Analytical laboratories

There is very little information available to the end user on laboratory size, numbers of personnel employed, types and distribution of instruments etc. One source of data is from surveys conducted by scientific journals [1], but these are naturally biased towards obtaining advertising revenue and are not specifically designed to provide information to the end user. Specialist marketing agencies also conduct surveys on a multi-client basis, but these are expensive and are never generally published. Similarly, major manufacturers also conduct private surveys: publication in this case is only likely to occur when some publicity gain is indicated, for example product X is used by more people than is its competitors.

The decision to install LIMS is probably the most important decision now faced by the laboratory manager. Unless based upon sound data about the industry, decision-taking becomes more of a lottery. It is obviously more desirable to make decisions based on fact, rather than to be swept along by market forces, gossip, or, even worse, because its fashionable. To aid this decision, a survey, specifically seeking information on current laboratory functions, was made. The ultimate objective was to relate laboratory functions to the costs of installing LIM systems. The survey was conducted from the Manchester Business School, as a part requirement of the part-time equivalent of the MBA degree.

### Questionnaire

All questions were condensed onto a single sheet, with multi-choice answers designed to minimize the time involved to about 15 min. Laboratory managers could most easily answer the questions. The recipients were selected randomly from the UK-based Chromatographic Society membership list, and was restricted to British members only [2]. A total of 121 questionnaires were released. Each recipient was previously contacted by telephone. Whilst members of the Society tend to be of senior status, it was anticipated that, where necessary, recipients would refer to a higher authority. Some 50% of the total questionnaires mailed were returned within three weeks. A second telephone prompt resulted in a further 20% being returned, and a third telephone call, a further 10%. Table 1 provides the returns data:

*Table 1. Returns.*

| | |
|---|---|
| 121 | questionnaires were sent out |
| 1 | recipient did not have any major instruments |
| 3 | were completed and returned but not received |
| 10 | replied with a blank form and apology, principally due to non relevance to their circumstances |
| 14 | did not reply |
| 91 | replies received. |

### *Market size*

The relevance of sample size to actual market size is of importance. The UK has 3542 sites designated as research laboratories [3], and the USA 50 705 [4]. It is highly probable that most of these sites will contain an analytical function in some form, but many will not conform to the distribution of instruments in the sample, since the emphasis in this survey is primarily towards carbon-based analytical systems. Other locations not so specified may of course use instruments in a non-research sense: for quality control, plant control etc. The sample of 91 laboratories represents less than 3% of the total designated as UK research laboratories. Consequently, extrapolation in order to estimate, for example the total number of instruments in the UK (81 700 of which 32 680 are gas chromatographs), and in the USA $1{\cdot}19 \times 10^6$ and $4{\cdot}7 \times 10^5$ respectively, should not be regarded as a reasonable method of estimating total numbers. Regrettably, however, the dearth of alternative data will almost certainly guarantee these data becoming enshrined in folklore and ultimately appearing as 'industry statistics'.

*Instrument types and distribution*

A total of 2099 major instruments were defined in the returns, of which 1552 were centralized, i.e. under the control of a single manager. The remainder (547) were on site but not centralized or directly controlled by the analytical manager. Thus the 'average' centralized laboratory has 17 major instruments installed, with a total of 23 on site. However, of the 10 blank forms returned with apologies (table 1), four were large laboratories, containing more than 50 instruments, and of which one was known to have more than 300 instruments. Consequently, all 'averages' will be an underestimate of the sample, and instrument distributions are therefore more relevant. Table 2 contains total numbers and percentages of specified instrument types. Where an instrument was mentioned less than six times (less than 0·4% of total sample), it was categorized under 'others'. Six techniques (gas chromatography, high-pressure liquid chromatography, ultra-violet, infra-red and atomic absorption spectroscopy and autoanalyser techniques totalled 86·6% of all).

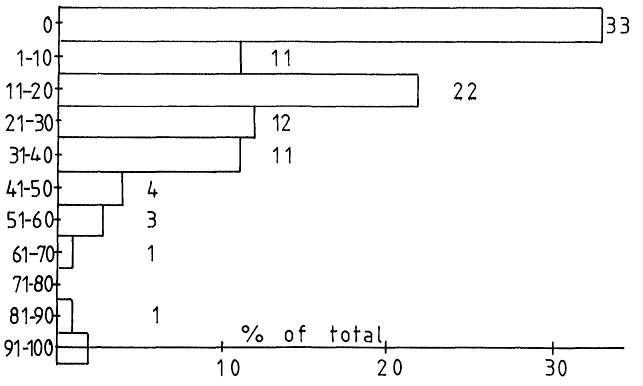

*Figure 2. Distribution of instruments by type (1) – HP liquid chromatographs.*

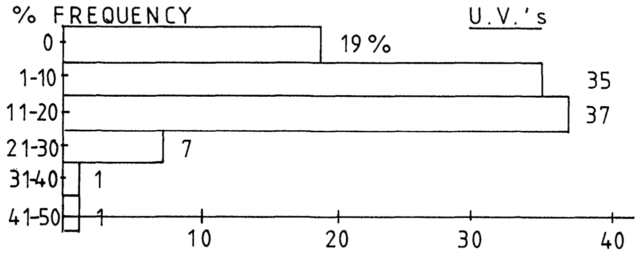

*Figure 3. Distribution of instruments by type (2) – UVs.*

*Table 2. Numbers and proportions of instruments.*

| | AA | Autoanalysis | Elem. analysis | GC | HPLC | ICP | MS | NMR | UV | XRF/XRD | TSA/DSC | SEM | Sorption analysis | αparticle spectr. | FIA | Auto titrators | *Other (<6) | IR | Total |
|---|---|---|---|---|---|---|---|---|---|---|---|---|---|---|---|---|---|---|---|
| No. | 94 | 77 | 27 | 622 | 313 | 9 | 41 | 32 | 139 | 28 | 14 | 10 | 6 | 8 | 6 | 10 | 19 | 97 | 155 |
| % | 6·1 | 5·0 | 1·7 | 40·0 | 20·2 | 0·6 | 2·1 | 2·1 | 9·0 | 1·8 | 0·9 | 0·6 | 0·4 | 0·5 | 0·4 | 0·6 | 1·2 | 6·3 | 10 |

The number of instruments ranged from 0–210. Distribution for centralized laboratories are given in table 3, and for non-centralized laboratories in table 4.

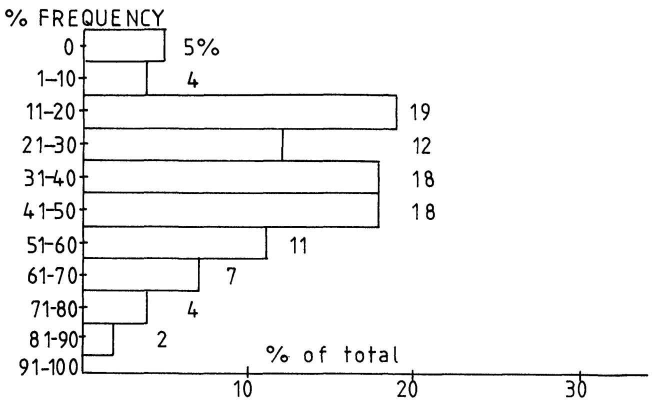

*Figure 1. Distribution of instruments by type (1) – gas chromatographs.*

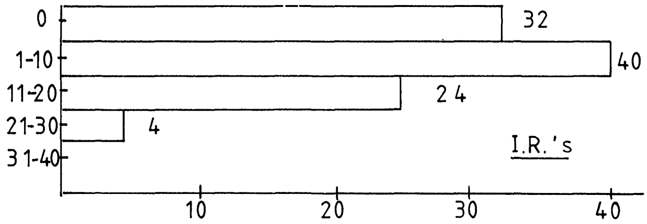

*Figure 4. Distribution of instruments by type (2) – IRs.*

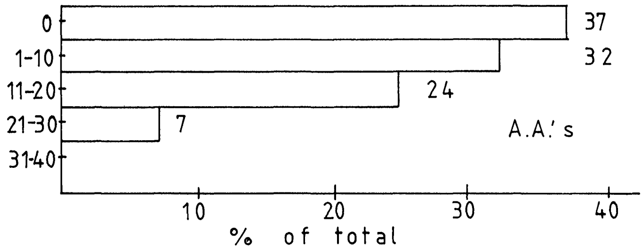

*Figure 5. Distribution of instruments by type (2) – AAs.*

*Table 3. Centralized laboratories.*

| | | | |
|---|---|---|---|
| 7% | had | 0 | instruments |
| 10% | ” | 1–5 | ” |
| 28% | ” | 6–10 | ” |
| 23% | ” | 11–15 | ” |
| 11% | ” | 16–20 | ” |
| 7% | ” | 21–25 | ” |
| 5% | ” | 26–30 | ” |
| 2% | ” | 31–35 | ” |
| 1% | ” | 36–40 | ” |
| 1% | ” | 41–45 | ” |
| 1% | ” | 46–50 | ” |
| 2% | ” | 51–55 | ” |
| 1% | ” | 126–130 | ” |
| 1% | ” | 201–210 | ” |

*Table 4. Non-centralized laboratories.*

| | | | |
|---|---|---|---|
| 42% | had | 0 | instruments |
| 22% | ” | 1–5 | ” |
| 17% | ” | 6–10 | ” |
| 5% | ” | 11–15 | ” |
| 4% | ” | 16–29 | ” |
| 4% | ” | 21–25 | ” |
| 1% | ” | 26–30 | ” |
| 1% | ” | 31–35 | ” |
| 1% | ” | 36–40 | ” |
| 1% | ” | 41–45 | ” |
| 1% | ” | 46–50 | ” |

Figures 1–5 show the distributions of the first five major instrumental techniques.

### Installed methodology

*Staff*

Table 5 shows the numbers and types of personnel employed, with distributions indicated in figure 6. It was not clear from the survey whether these staff were exclusively employed in the centralized unit, or whether they also operated the non-centralized units. It has been assumed that the total 2033 employees stated operated all 2099 instruments.

An attempt was made to correlate the distribution of employees with numbers of instruments; some laboratories had as few as one person per three/four instruments, others exceeded five employees per instrument. The highest ratio laboratories were checked, and confirmation received that considerable work other than purely analytical was also conducted. Conversely, the 'efficient' laboratory tended to concentrate entirely upon analytical operations. Nevertheless, most laboratories appeared to be regarded as a purely analytical laboratory by the remainder of the company. The statistics also indicated that the larger the laboratory, the lower the percentage of graduates employed.

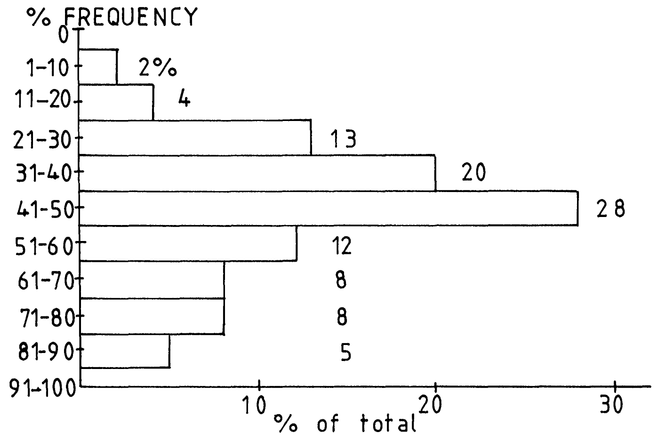

*Figure 6. Distribution of personnel (graduates and equivalents).*

*Table 5. Number and status of personnel employed.*

| | |
|---|---|
| Graduates and equivalent | 890 (44%) |
| Technicians | 852 (42%) |
| Others | 291 (14%) |
| Total | 2033 |

*Data inputs and reporting*

These were classified as outputs by instruments with an integral microprocessor (MCP) or computer, those operating through an analogue to digital convertor with printer output, and finally those without any digital outputs, classified as manually processed. Tables 6 and 7 provide percentage data for centralized and non-centralized instruments respectively. No relationship could be established between the percentage of MCP controlled instruments as a function of the number of instruments in the specified location. In terms of distribution, 1% of centralized laboratories had 100% manually processed outputs, whereas 7% had 100% MCP outputs; for non-centralized laboratories, 20% had 100% manually processed outputs, with 21% having 100% MCP outputs.

*Table 6. Centralized laboratories.*

| | % of total instruments |
|---|---|
| Manual processing | 31 |
| Printer | 19 |
| MCP | 50 |

*Table 7. Non-centralized laboratories.*

| | % of total instruments |
|---|---|
| Manual processing | 66 |
| Printer | 23 |
| MCP | 11 |

Data transmission methods were reported as shown in table 8.

*Table 8. Data transmission methods.*

| Method | % of total |
|---|---|
| Directly by operator (verbally) | 27 |
| By standard written report | 50 |
| By standard written report and attached print-out | 23 |

*Popularity of named manufacturers*

Where instruments had been data linked, the name of the equipment manufacturer was requested. Only six manufacturers were named more than once. Those mentioned once only in the total sample were not included. The popularity of the six are described in table 9 as a percentage of total systems installed. It was also possible to relate the named manufacturers with the average number of instruments in the laboratory, the average number of GCs and HPLCs available for linking in the laboratory, and the percentage of those available which were actually linked.

*Table 9. Popularity of named manufacturers.*

| | % of named manufacturers |
|---|---|
| Trivector | 27 |
| Perkin Elmer | 26 |
| Hewlett-Packard | 21 |
| Spectra Physics | 11 |
| VG* | 10 |
| DEC | 5 |

* Note that VG systems are based on DEC CPUs.

*Table 10. Relationship to instruments in use.*

| | Average number of instruments in the lab. | Average number of GCs/HPLCs in the lab. | Average % of GCs/HPLCs linked |
|---|---|---|---|
| Trivector | 16 | 11 | 71 |
| Perkin Elmer | 13 | 8 | 43 |
| Hewlett-Packard | 26 | 16 | 59 |
| Spectra Physics | 24 | 18 | 32 |
| VG | 24 | 15 | 47 |
| DEC* | 27 | 12 | 75 |

* Small sample may distort the results.

49% of centralized laboratories have installed at least one named data linking system, but only 39% of all GC/HPLCs available were operating through a CPU, and, on average, only six instruments were linked. There appears to be considerable further scope for the data linking of instruments to an already purchased CPU.

*Assessment of efficiency*

Managers and operators were asked for their views of their data-handling methods. 53% of instrument operators were satisfied with their methods, but 57% of managers were not, dissatisfaction increasing as the number of instruments under their control increased. Some 81% of managers were involved in interpretation, writing and checking of reports, whilst 73% felt that efficiency could be improved. Despite the high proportion stating that efficiency could be improved, some 68% of the total sample had not quantitatively evaluated the reasons for the efficiency shortfall. Upon prompting, the results of the scale of the efficiency problem are given in figure 7. Managers felt that efficiency was reduced for the reasons given in table 11.

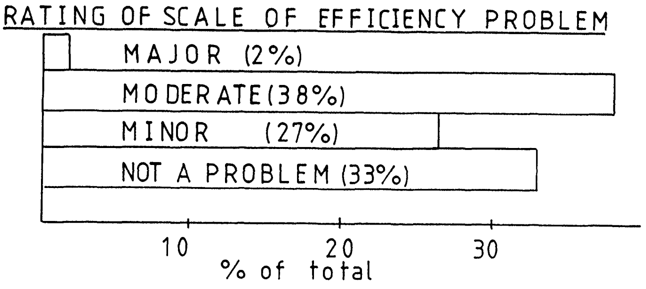

*Figure 7. Rating of scale of efficiency problem.*

*Table 11. Reasons for efficiency reduction.*

| | % of total |
|---|---|
| Fewer samples can be processed per unit time | 29 |
| Inefficient use of manager's time | 23 |
| Poorer quality of data | 18 |
| Staff dissatisfaction with repetitive work | 16 |
| Cannot use 24 h cycles | 14 |

With multiple answers = 59%
With single answers = 31%
With no answer despite stating efficiency could be improved = 10%

*Price managers prepared to pay*

Finally, recipients were asked whether they had estimated the price they were prepared to pay to overcome their lack of efficiency; it emerged that 71% had not yet made this assessment. This is not too surprising, since LIMS is conceptually very new, and very little information has been published. In addition, from a manager's position, research functions are notoriously difficult to cost. Accountants normally compile departmental costs, and impose them on the manager, few managers appear to agree with their derivation.

Although a very high proportion of recipients had not actually estimated costs, most were prepared to estimate a level of proposed expenditure. These are given in table 12.

*Table 12. Capital sum prepared to spend.*

| | % of total |
|---|---|
| Nothing | 28 |
| £10 000 | 18 |
| £25 000 | 28 |
| £50 000 | 13 |
| £75 000 | 5 |
| £100 000 | 5 |
| Over £100 000 | 3 |

However, absolute costs are of little relevance unless related to the number of instruments in the laboratory. Price per channel was calculated by the simple expedient of dividing the price that managers were prepared to pay by the total number of instruments in the centralized laboratory (table 13).

*Table 13. Cost per channel.*

| | % |
|---|---|
| Prepared to pay less than £1500 per channel | 31 |
| Prepared to pay £1500–£3500 | 36 |
| Prepared to pay £3500–£5000 | 13 |
| Prepared to pay more than £5000 | 20 |

However, the cost per channel indicated may be an underestimate of the recipients' intentions. It is probable that at this early stage of LIMS development most managers are considering only linking the most commonly used instruments. Also, only 50% of existing instruments are MCP controlled. A second source of possible error is that the cost of purchasing the basic CPU and peripherals is very high compared to the investment cost of adding one extra channel, and for those laboratories with less than five instruments the cost per channel would be exceedingly high and therefore underestimated by the manager. However, since 83% of the sample have more than six instruments, the basic initial costs should not be a major deterrant.

### Conclusions

The data presented here can be used to develop advantages, and costs, of installing LIM systems.

### References

1. *International Laboratory, Laboratory Survey* (1983).
2. The Chromatographic Society, c/o Trent Polytechnic, Burton Street, Nottingham NG1 4BU, UK.
3. *Industrial Research in the UK* (10th edn, Longman, London, 1983).
4. American Laboratory, ISC, Woodside Road, Amersham, Bucks, UK (1984).

**35th CANADIAN CHEMICAL ENGINEERING CONFERENCE**

*Calgary, Alberta, 6–9 October 1985*

Organized by the Canadian Society for Chemical Engineering/Sociêté Canadienne du Génie Chimique, the conference will be held at the Calgary Convention Centre, and will consist of seven concurrent technical and general-interest sessions. The papers will cover a wide range of topics from fundamentals to industrial applications of chemical engineering. There will also be sessions relevant to the chemical, process, and energy industries. Several sessions, including one on government relations, will include invited speakers. The economic and Business Management Division (EBM) of the Chemical Institute of Canada is co-sponsoring and organizing several sessions on forecasts, forecasting and planning, petrochemicals, and the business side of large projects.

Technical sessions at the conference are planned on the following subjects:

Biotechnology
Business side of large projects (EBM)
Chemical engineering fundamentals with applications
Chemical processing
Coal, oil and tar sands
Cogeneration
Computer-aided design
Computer control
Entrepreneurs in chemical engineering
Environmental opportunities
Environmental regulations
Forecasts, forecasting and planning (EBM)
Government relations
Petrochemical outlook (EBM)
Plastics and materials
The gas plant industry
Use of PCs in chemical engineering
Utilization of methane.

*Further information from Roger M. Butler, Department of Chemical and Petroleum Engineering, University of Calgary, Calgary, Alberta T2N 1N4, Canada. Tel.: 403 284 7133.*

